# Molecular Characterization of an Isolate of Tobacco Streak Virus Naturally Infecting *Areca catechu* L. in China—A First Case in the Family Arecaceae

**DOI:** 10.3390/plants15121864

**Published:** 2026-06-16

**Authors:** Xupeng Wang, Qingjuan Wu, Lingmin Zou, Xiaoqi Jiang, Zhen Li, Wei Hu, Shuli Xian, Xueyuan Xia, Zhongguo Xiong, Naitong Yu, Yuliang Zhang

**Affiliations:** 1 Institute of Tropical Bioscience and Biotechnology, Chinese Academy of Tropical Agricultural Sciences, Haikou 571101, China; wangxupeng@catasitbb.cn (X.W.);; 2 Key Laboratory of Nanfan Biotech Breeding, Ministry of Agriculture and Rural Affairs, Sanya Research Institute, Chinese Academy of Tropical Agricultural Sciences, Sanya 572525, China; 3 College of Plant Protection, Nanjing Agricultural University, Nanjing 210095, China; 4 State Key Laboratory of Crop Stress Adaptation and Improvement, School of Life Sciences, Henan University, Kaifeng 475004, China; 5 School of Plant Sciences and BIO5 Institute, University of Arizona, Tucson, AZ 85721, USA

**Keywords:** tobacco streak virus, *Areca catechu* L., natural infection, genome sequence

## Abstract

Tobacco streak virus (TSV) has a wide range of natural host plants, yet it has not been detected in plants of the Arecaceae family. In Hainan, China, areca plants exhibited yellow-green chlorotic streaked leaves, and irregular chlorotic patches appeared on the main stem, which collectively represents typical TSV-infection symptoms. In this study, the near-complete genome sequence of the TSV A6-5 isolate was obtained through meta-transcriptome sequencing combined with reverse transcription polymerase chain reaction (RT-PCR). Molecular characterization indicated that its RNA1 (3435 nt), RNA2 (2841 nt), and RNA3 (2193 nt) shared 99.27%, 99.16% and 98.40% nucleotide sequence identity, respectively, with the previously documented TSV DSMZ PV-0612 isolate from *Rudbeckia* sp. in Germany. No recombination signals were detected in the RNA1, RNA2, and RNA3 sequences of the TSV A6-5 isolate using all algorithms embedded in RDP5, indicating the isolate is highly evolutionarily conserved. Furthermore, phylogenetic analyses based on the full-length sequences of RNA1, RNA2 and RNA3 collectively verified that the TSV A6-5 is most closely related to TSV DSMZ PV-0612. This study documents the first case of natural TSV infection in *Areca catechu* L. worldwide and provides a theoretical foundation for the monitoring and control of TSV in areca palm production.

## 1. Introduction

Areca palm (*Areca catechu* L.), a perennial commercial crop in the family Arecaceae, is widely cultivated across tropical Asia with prominent economic value, and its fruits are mainly utilized for fresh chewing consumption and as raw material for traditional Chinese medicine. In recent years, viral diseases have become a major limiting factor restricting the healthy production of areca palm. To date, multiple viral pathogens infecting areca palm have been identified, including members of the genera *Velarivirus* [1], *Arepavirus* [2,3], and *Totivirus* [4]. Nevertheless, systematic information regarding the viral diversity associated with areca palm and the economic impacts of these viral pathogens remains poorly characterized.

Tobacco streak virus (TSV, *Ilarvirus TSV*), a member of the genus *Ilarvirus* in the family *Bromoviridae*, possesses an extremely wide host spectrum and is capable of infecting over 200 plant species from more than 30 dicot and monocot families [5,6]. As a multipartite, single-stranded, positive-sense RNA virus, its genome comprises three genomic RNAs: RNA1 encodes protein 1a; RNA2 encodes 2a (RNA-dependent RNA polymerase, RdRP) and 2b; and RNA3 encodes movement protein (MP) and coat protein (CP) [6]. TSV affects numerous economically important crops such as tobacco, cotton, peanut, soybean, sunflower, and cucurbit vegetables [7,8,9,10,11,12], causing severe yield and quality losses in many countries worldwide. TSV can be transmitted through multiple routes, including mechanical inoculation, pollen, seeds, and thrips, and tends to be more epidemic under hot and humid tropical conditions [13,14]. Despite its wide host range, diverse transmission modes, and complex genetic variation, no natural infection of TSV in plants of the family Arecaceae has been formally reported globally to date, and its potential threat to economic palm crops including areca palm has long been overlooked.

In June 2025, during a field survey in the main areca palm-producing areas of Sanya, Hainan, China, our research group observed viral disease-like symptoms on an areca palm plant, including yellow-green chlorotic streaks on leaves and irregular chlorotic patches on stems. To identify the causal pathogen, meta-transcriptome sequencing combined with RT-PCR was used for pathogen detection and whole-genome sequence analysis in this study. The results confirmed for the first time that TSV can naturally infect areca palm, representing the first global record of natural TSV infection in the family Arecaceae. This study not only enriches the data on the host range and geographical distribution of TSV but also provides important materials for further understanding the host adaptation, cross-host transmission, and genetic evolution of TSV, as well as a theoretical basis for the monitoring, early warning, and green control of TSV in areca palm-producing areas of China.

## 2. Results and Discussion

### 2.1. Field Disease Survey and Molecular Detection

**Figure 1:**
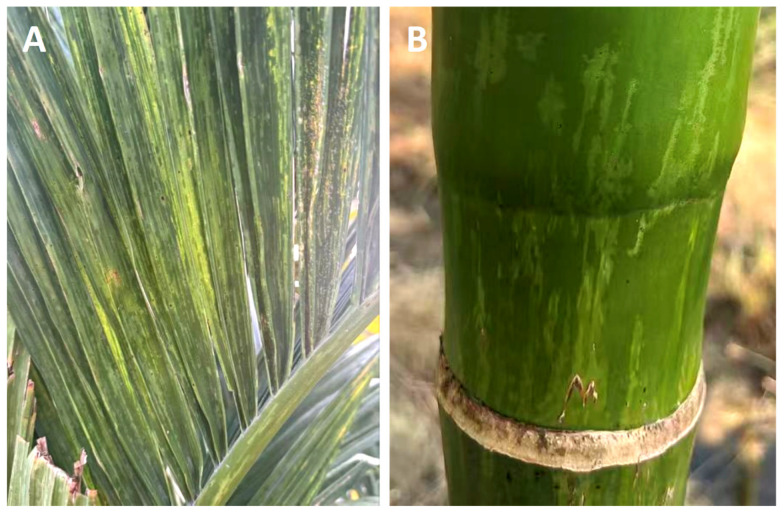
Symptoms of suspected viral infection in naturally infected areca palm plants. (**A**) Infected leaves showed prominent yellow-green chlorotic streaks, (**B**) while irregular chlorotic patches were observed on the main stem, collectively presenting typical mosaic symptoms.

In June 2025, a field disease survey was conducted in areca palm-growing areas covering 12 administrative villages in Yazhou District, Sanya City, Hainan Province, China. A total of 93 leaf samples were collected during the investigation. Among these samples, A6-5 exhibited typical viral infection symptoms, characterized by prominent yellow-green chlorotic streaks on the leaves and irregular chlorotic patches on the main stem (Figure 1). RT-PCR results further confirmed that only sample A6-5 was infected with TSV using the primer pair TSV-2077-RNA2-F4/TSV-2855-RNA2-R4.

### 2.2. Genome Assembly, Validation and Sequence Determination of TSV Isolate A6-5

Viral genome assembly results showed that the assembled lengths of RNA1, RNA2 and RNA3 for the TSV A6-5 isolate were 3397 nt (with a 31 nt gap), 2798 nt (with a 40 nt gap), and 2196 nt (with a 40 nt gap), respectively (Supplementary File S1). To verify the assembled sequences and fill the internal gaps, specific primers covering the full length of each genomic segment were designed, and the corresponding amplicons were cloned and subjected to Sanger sequencing. The near-complete tripartite genome of TSV isolate A6-5 was finally determined by integrating meta-transcriptomic data with RT-PCR gap-filling results. The genome sizes of RNA1, RNA2, and RNA3 were 3435 nt, 2841 nt, and 2193 nt, respectively. The genome sequences of TSV A6-5 isolate have been deposited in the GenBank database under the accession numbers PZ244272 (RNA1), PZ244273 (RNA2) and PZ244274 (RNA3).

### 2.3. Nucleotide Sequence Identity and Phylogenetic Analysis of TSV A6-5

Among all publicly available TSVs, the A6-5 isolate shared the highest nucleotide sequence similarity with the German isolate DSMZ PV-0612 derived from *Rudbeckia* sp., with sequence identities of 99.27% (RNA1), 99.16% (RNA2), and 98.40% (RNA3), respectively. The A6-5 isolate showed relatively consistent sequence similarity to other TSV isolates in RNA1 and RNA2, whereas obvious divergence was observed in RNA3 (Table 1). These results suggest that potential recombination events may have occurred in the RNA3 sequence.

Recombination analysis performed using RDP5 software (v.5.84) revealed no recombination events in the RNA1, RNA2 and RNA3 sequences of isolate A6-5 when compared with 31 TSV isolates. Five TSV isolates with the closest genetic relationship to A6-5 were subsequently selected for pairwise RDP comparison analysis. No inter-isolate recombination was detected in the RNA1 and RNA2 sequences of these TSVs, whereas distinct recombination signals were identified in their RNA3 sequences (Table 2). Isolates including dp, IA-3-2017 and Henry exhibited this recombination feature (Figure S1). These findings demonstrate that the genome of TSV isolate A6-5 is highly evolutionarily conserved.

**Figure 2:**
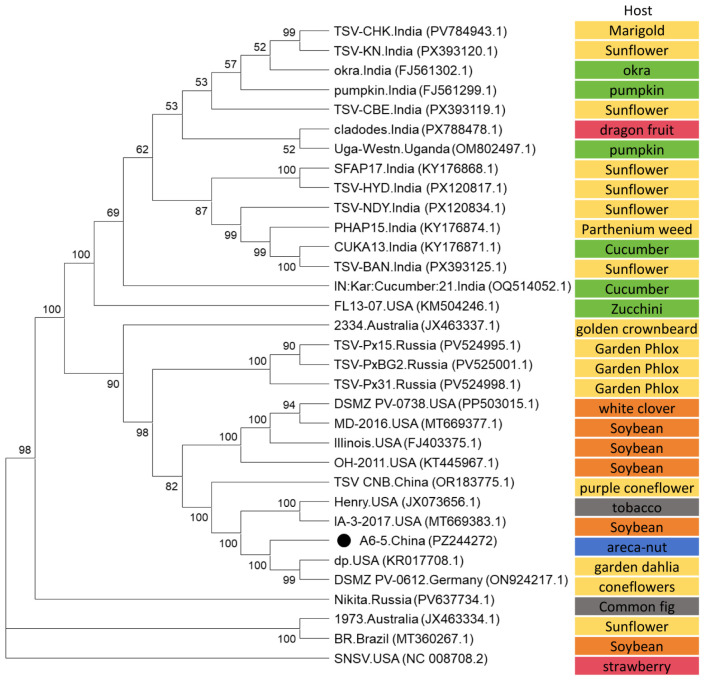
Phylogenetic tree constructed based on the full-length nucleotide sequences of TSV RNA1 using the Neighbor-Joining algorithm. Branch colors represent different host families: yellow for Asteraceae (sunflower, coneflowers, garden dahlia, marigold, golden crownbeard, purple coneflower, garden phlox, parthenium weed); green for Cucurbitaceae (cucumber, zucchini, pumpkin); orange for Fabaceae (white clover, soybean); red for Rosaceae (dragon fruit, strawberry); blue for Arecaceae (areca nut). Strawberry necrotic shock virus (SNSV), a member of the genus *Ilarvirus*, was used as the outgroup.

**Figure 3:**
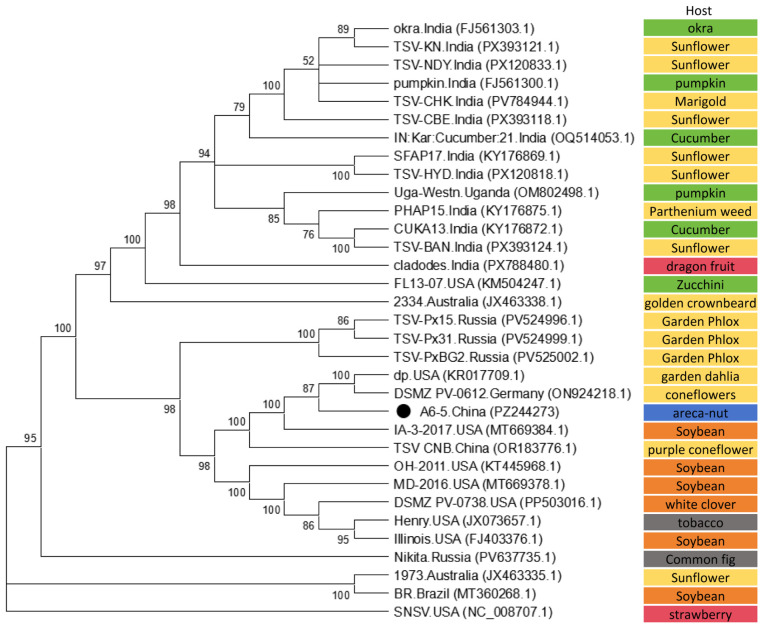
Phylogenetic tree constructed based on the full-length nucleotide sequences of TSV RNA2 using the Neighbor-Joining algorithm. Branch colors represent different host families: yellow for Asteraceae (sunflower, coneflowers, garden dahlia, marigold, golden crownbeard, purple coneflower, garden phlox, parthenium weed); green for Cucurbitaceae (cucumber, zucchini, pumpkin); orange for Fabaceae (white clover, soybean); red for Rosaceae (dragon fruit, strawberry); blue for Arecaceae (areca nut). SNSV was used as the outgroup.

**Figure 4:**
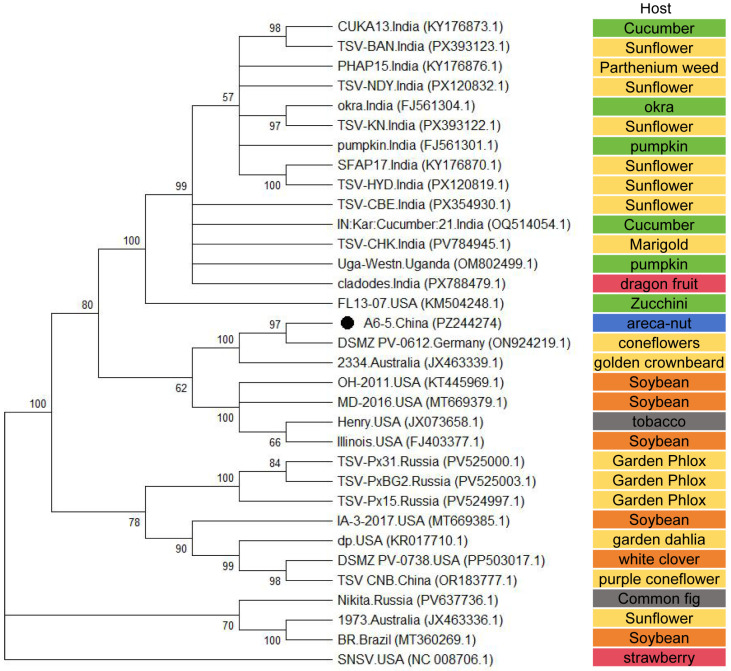
Phylogenetic tree constructed based on the full-length nucleotide sequences of TSV RNA3 using the Neighbor-Joining algorithm. Branch colors represent different host families: yellow for Asteraceae (sunflower, coneflowers, garden dahlia, marigold, golden crownbeard, purple coneflower, garden phlox, parthenium weed); green for Cucurbitaceae (cucumber, zucchini, pumpkin); orange for Fabaceae (white clover, soybean); red for Rosaceae (dragon fruit, strawberry); blue for Arecaceae (areca nut). SNSV was used as the outgroup.

In the phylogenetic tree constructed based on the full-length nucleotide sequences of RNA1, the TSV isolate A6-5 derived from *Areca catechu* formed a tight cluster with American strains, exhibiting high genetic relatedness and being clearly assigned to the American lineage. In contrast, it showed a large evolutionary distance from isolates from Eurasia and South Asia, presenting typical evolutionary characteristics of the American lineage (Figure 2). Similarly, consistent results were obtained from the phylogenetic tree based on the RNA2 (Figure 3). In the RNA3-based phylogenetic tree, the phylogenetic placement of the A6-5 isolate was significantly shifted: although the isolate still maintained relatively close genetic relatedness with some TSV strains of European and American origin, its overall topological assignment was distinctly different from that in the RNA1- and RNA2-based trees (Figure 4), showing clear signals of recombination. This result is consistent with the conclusion from the previous RDP recombination analysis that the RNA3 segment of TSV is prone to recombination and also indirectly suggests that the RNA3 segment has a relatively higher evolutionary rate and is more susceptible to genetic variation through recombination events within the American lineage.

### 2.4. Mechanical Inoculation Assay of Areca Palm Leaves

Friction inoculation was performed using naturally infected areca palm leaves. A supplementary inoculation was performed one month after the initial treatment. The results showed that no typical viral symptoms were observed on any inoculated areca palm seedlings at 3–4 weeks post inoculation. Moreover, no specific fragments of TSV were detected by RT-PCR assay. This failure to establish effective infection was probably attributed to the low viral titer in the inoculum and the thick cuticle of areca palm leaves, which prevented viral penetration via simple surface abrasion alone.

## 3. Materials and Methods

### 3.1. Total RNA Extraction, Meta-Transcriptomic Sequencing and Data Deposition

To identify the causal virus, total RNA was extracted from pooled leaf tissues using the TRIzol^TM^ reagent kit (Invitrogen^TM^) and subjected to meta-transcriptomic sequencing at Shanghai Biozeron Biotechnology Co., Ltd. (Shanghai, China). A transcriptome library was constructed using the TruSeq™ RNA Sample Prep Kit (Illumina) and sequenced on the Illumina HiSeq platform with 150 bp paired-end reads. A total of 278,790,556 raw reads were generated. Low-quality reads, adapter sequences, and sequences containing poly(A) or poly(N) stretches were filtered out using Trimmomatic software (version 0.36), yielding 267,760,184 clean reads. The raw sequencing data have been deposited in the Genome Sequence Archive (GenBase) [15] at the National Genomics Data Center [16], Beijing Institute of Genomics, Chinese Academy of Sciences/China National Center for Bioinformation, under accession number CRA034921, and are publicly accessible at https://ngdc.cncb.ac.cn/genbase (accessed on 14 June 2026).

### 3.2. Sequence Assembly, Annotation and Preliminary Identification of TSV

The obtained clean reads were *de novo* assembled using Velvet software (version 1.2.10) with a k-mer value of 17. The assemble contigs were further annotated via local BLASTn and BLASTx searches against a viral reference database downloaded from NCBI (ftp://ftp.ncbi.nlm.nih.gov/refseq/release/viral/) (accessed on 13 January 2026). Multiple viral contigs shared high sequence similarity with tobacco streak virus (TSV). To recover the nearly complete genome sequence of the TSV isolate A6-5, the full-length RNA1 (ON924217.1), RNA2 (ON924218.1), and RNA3 (ON924219.1) sequences of the closely related TSV isolate DSMZ PV-0612 retrieved from the NCBI GenBank database were used as reference templates for genome assembly. Viral read mapping, genome assembly, and pathogen identification were performed following the bioinformatics pipeline described by Zhang et al. [17].

### 3.3. RT-PCR Assay of TSV

To validate the assembled sequences and fill the internal gaps, specific primers spanning the full length of each genomic segment were designed based on the assembled contigs and the published genome of TSV isolate DSMZ PV-0612 (Table 3). Total RNA extracted from the diseased original sample was reverse-transcribed to cDNA using the HiFiScript cDNA Synthesis Kit (CWBIO Biotech, Beijing, China). PCR amplification was then carried out with 2×SanTaq PCR MasterMix (Sangon Biotech, Shanghai, China). All reaction systems and experimental operations followed the manufacturer’s recommended protocols. Target amplicons of expected size were successfully obtained for all three genomic segments, cloned into vectors, and subjected to Sanger sequencing by Shanghai Shenggong Biotechnology Co., Ltd.

### 3.4. Phylogenetic Trees Constructed Based on the Full-Length Nucleotide Sequences of TSV RNA1, RNA2 and RNA3

Nucleotide sequence identity analysis was performed on the three genomic segments of TSV isolate A6-5 on Genbank database in NCBI. Recombination analysis was conducted using nine methods (RDP, GeneConv, Bootscan, MaxChi, Chimaera, SiScan, 3Seq, Lard, and Phylpro) implemented in RDP5 (v.5.84) [18]. A recombination event was considered reliable if it was detected by at least three of the nine methods with a *p*-value < 0.05.

Phylogenetic trees constructed based on the full-length nucleotide sequences of TSV RNA1, RNA2 and RNA3 using the Neighbor-Joining algorithm (Table S1). Bootstrap values (1000 replicates) are shown at each branch node. Evolutionary distances were calculated using the Kimura 2-parameter model and represented as the number of nucleotide substitutions per site. The phylogenetic analysis included 33 nucleotide sequences, and ambiguous positions were removed by pairwise deletion, resulting in a final alignment of 3570 (RNA1), 3043 (RNA2) and 2333 (RNA3) nucleotide positions. All phylogenetic analyses were performed in MEGA 12 (version 12.1.2) using up to seven parallel computing threads [19,20].

### 3.5. Mechanical Inoculation Method

Mechanical inoculation assays were carried out using crude sap extracted from naturally infected areca palm leaves. Leaf tissues were homogenized in 0.01 M phosphate buffer (pH 7.2) at a 1:10 g/mL tissue-buffer ratio, and the obtained sap was mechanically inoculated onto leaves of 30 areca palm seedlings pre-dusted with carborundum powder. A supplementary inoculation was conducted one month after the first treatment. All inoculated seedlings were maintained under field conditions in Sanya, Hainan, China.

## 4. Conclusions

This study reports the first global case of natural infection by TSV in *Areca catechu* L., representing a novel host record in the Arecaceae family. The near-complete tripartite genome of the TSV isolate A6-5 from areca palm was obtained via meta-transcriptome sequencing combined with RT-PCR. Sequence identity analysis revealed that the A6-5 isolate shares the highest nucleotide similarity with the German isolate DSMZ PV-0612, with identities of 99.27% (RNA1), 99.16% (RNA2), and 98.40% (RNA3).

Phylogenetic analyses showed that the A6-5 isolate clusters stably within the American lineage in the RNA1 and RNA2 trees, while its phylogenetic position shifts noticeably in the RNA3 tree, displaying distinct topological incongruence. RDP5 confirmed that no recombination signals exist in RNA1, RNA2 and RNA3 of the A6-5 isolate, whereas RNA3 of several closely related TSV isolates exhibits obvious recombination events. These findings collectively demonstrate that the TSV RNA3 segment derived from other hosts is prone to genetic recombination and has a relatively higher evolutionary rate.

As it reports on the first TSV isolate naturally infecting areca palm worldwide, this study not only expands the known host range and geographical distribution of TSV but also provides critical molecular evidence for the virus’s capacity for long-distance dispersal, genetic recombination, and host jumping. This study lays a theoretical foundation for surveillance, early warning, and sustainable management of TSV in areca palm-growing regions and also offers valuable insights into the evolutionary adaptation and cross-host transmission mechanisms of TSV.

## Figures and Tables

**Table 1:** Nucleotide sequence identity (%) between A6-5 isolate and other closely related TSV isolates.

Isolates	RNA1	RNA 2	RNA 3
DSMZ PV-0612	99.27%	99.16%	98.63%
dp	99.36%	99.23%	92.75%
IA-3-2017	97.82%	97.85%	91.90%
Henry	97.76%	87.38%	92.31%
2334	89.89%	88.53%	96.58%

**Table 2:** Recombination analysis of RNA1, RNA2 and RNA3 sequences between TSV isolate A6-5 and other closely related isolates using RDP5.

Isolates	RNA1	RNA2	RNA3
A6-5	No	No *	No
DSMZ PV-0612	No	No	No
dp	No	No	Yes
IA-3-2017	No	No	Yes
Henry	No	No	Yes
2334	No	No *	No

“*” indicates a potential recombination signal, but its reliability in the software analysis is low.

**Table 3:** Complete list of oligonucleotide primers used in this study.

Primer Name	Primer Sequence (5′-3′)	Targeted Region
TSV-12-RNA1-F1	TTGTATTCGAATCAGAACCTCC	RNA1, 1300 bp
TSV-1311-RNA1-R1	CAGCTAGTGGAACATATTCATCG	
TSV-1056-RNA1-F2	GTCAAATTAGCCGTTCCTGTC	RNA1, 1383 bp
TSV-2438-RNA1-R2	GATCTTAAAGGTTGGGACCAGT	
TSV-2414-RNA1-F3-1	ATAAGTGGTACCCTTCTGCAC	RNA1, 1075 bp
TSV-3488-RNA1-R3	GCATCTCCTTTTAGGAGGCAT	
TSV-SY-311-RNA1-F	CTCTTGTCATTCGTCACACAGTTTT	RNA1, 1861 bp
TSV-SY-2171-RNA1-R	TAGGGCATCGTATGTTTGCTTATTC	
TSV-13-RNA2-F1	TGTATCCGAATCAGAACCTCC	RNA2, 1544 bp
TSV-1556-RNA2-R1	CAATCACGATCCCTAATGTGA	
TSV-1356-RNA2-F2-1	CCAGATATTCCAAATGAATCCCG	RNA2, 1019 bp
TSV-2374-RNA2-R2-1	CAACTCCACTGGATTGATTGT	
TSV-2317-RNA2-F3	CGGAGAGACCATAAACGTGAAGGCG	RNA2, 542 bp
TSV-2858-RNA2-R3	AATCAGTGGGAAACATAGAGAAGCG	
TSV-2077-RNA2-F4	TTAGTGGCGAACAGGATGAAAT	RNA2, 779 bp
TSV-2855-RNA2-R4	CAGTGGGAAACATAGAGAAGCG	
TSV-SY-620-RNA2-F	GTTTCGTTTCGTATGCCTCTGGGA	RNA2, 1746 bp
TSV-SY-2365-RNA2-R	TGGATTGATTGTATTGGGCTGTTC	
TSV-1-RNA3-F1	GTATTCTCCGAGCAAAGATACCA	RNA3, 1199 bp
TSV-1199-RNA3-R1	GTAAGTCCGAGAAGCGACTTAT	
TSV-893-RNA3-F2	GATATGCTCGGAACGTGTTAG	RNA3, 1317 bp
TSV-2209-RNA3-R2	GCATCTCCTAAAAGGAGGCATC	
TSV-SY-380-RNA3-F	GACAACCAAAGAGACGAAATCCT	RNA3, 1481 bp
TSV-SY-1860-RNA3-R	TCCAATAACCTGCCAGCTGAACC	

## Data Availability

The meta-transcriptomic data have been deposited in the GenBase in National Genomics Data Center, Beijing Institute of Genomics, Chinese Academy of Sciences/China National Center for Bioinformation, under accession number CRA034921. The genome of the TSV A6-5 isolate has been deposited in the GenBank database under accession numbers PZ244272 (RNA1), PZ244273 (RNA2), PZ244274 (RNA3).

## Supplementary Materials

The following supporting information can be downloaded at https://www.mdpi.com/article/10.3390/plants15121864/s1: Table S1: List of tobacco streak viruses and strawberry necrotic shock virus information from different plant host sources; Figure S1: RDP-based recombination analysis of TSV RNA3 genomic sequences; Supplementary File S1: The genome of TSV isolate A6-5 was assembled from meta-transcriptomic sequencing data.
