# Supplementary material for: Molecular Characterization of an Isolate of Tobacco Streak Virus Naturally Infecting Areca catechu L. in China—A First Case in the Family Arecaceae
Source: Plants (Basel). 2026 Jun 16;15(12):1864. doi: 10.3390/plants15121864 (PMC13306292; doi:10.3390/plants15121864)
Supplement: Supplementary file 1 [file plants-15-01864-s001.zip › Figure S1. RDP-based recombination analysis of TSV RNA3 genomic sequences.pdf]

(a) dp

Confirmation Table		
Methods	# seqs detected in	Av. P-Val
RDP	1	1.605 X 10^{-06}
GENECONV	1	1.245 X 10^{-05}
BootScan	1	1.245 X 10^{-05}
MaxChi	1	5.500 X 10^{-10}
Chimaera	1	3.291 X 10^{-06}
SiScan	1	4.184 X 10^{-08}
3Seq	1	6.269 X 10^{-09}
LARD	--	--
Phylpro	--	--

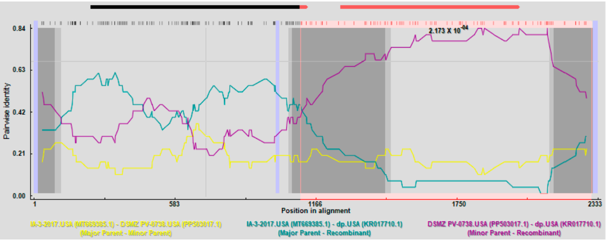

(b) IA-3-2017

Confirmation Table		
Methods	# seqs detected in	Av. P-Val
RDP	2	5.503 X 10^{-03}
GENECONV	--	--
BootScan	--	--
MaxChi	12	3.389 X 10^{-02}
Chimaera	4	1.072 X 10^{-02}
SiScan	8	1.709 X 10^{-13}
3Seq	--	--
LARD	--	--
Phylpro	--	--

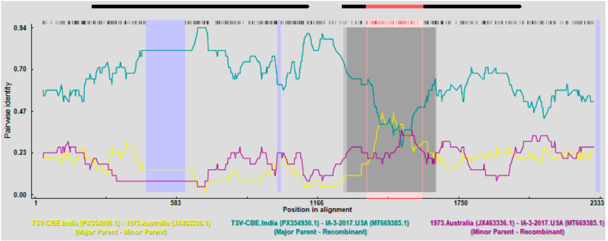

(c) Henry

Confirmation Table		
Methods	# seqs detected in	Av. P-Val
RDP	--	--
GENECONV	--	--
BootScan	--	--
MaxChi	10	6.304 X 10^{-03}
Chimaera	8	5.447 X 10^{-04}
SiScan	6	6.925 X 10^{-19}
3Seq	--	--
LARD	--	--
Phylpro	--	--

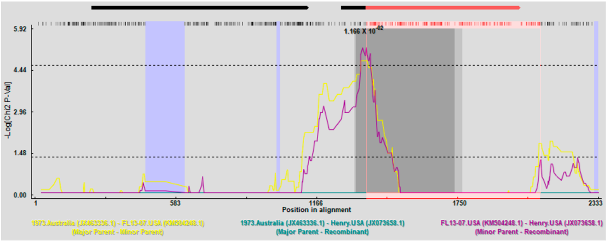

Figure S1. Detection of recombination events of RNA3 genomic sequences in TSV isolates (a) dp, (b) IA-3-2017, and (c) Henry, using nine algorithms (RDP, GeneConv, Bootscan, MaxChi, Chimaera, SiScan, 3Seq, Lard, and Phylpro) implemented in RDP5. Any recombination event predicted by at least three of the nine methods with a P-value < 0.05 was considered reliable and included in the analysis.
