# Supplementary material for: Molecular Characterization of an Isolate of Tobacco Streak Virus Naturally Infecting Areca catechu L. in China—A First Case in the Family Arecaceae
Source: Plants (Basel). 2026 Jun 16;15(12):1864. doi: 10.3390/plants15121864 (PMC13306292; doi:10.3390/plants15121864)
Supplement: Supplementary file 1 [file plants-15-01864-s001.zip › Supplementary file 1 The genome of TSV A6-5 was assembled from meta-transcriptomic sequencing data.pdf]

>TSV RNA1 (A6-5 isolate)
CCGAATCAGAACCTCCAGATAATGGATTCCTTATCTTTACCCACCGTGAGTGACGTTGTTGTTCCTGCTCTTAA
CGTTGACAATCTCATTCGAGATTATGTCAGTAACGTGAGAGCCGATGATAGCAATAACGTCAGTAGATTCCTC
GGTGAAGTAGCCTTGAAAGAGATTAAGTCTCAAGTTGACACCAGTAATGGTGATTTCCAGAAGTTGAACGT
CGGTTTTCGTTTGACTCCTGATGAAAAGAATGCGCTGAAGGCGAACTTTCCTGGTCTCGAGATTGCATTTAG
AGACTCTTGTCATTCGTCACACAGTTTTGCCGCAGCTCATCGAGTTTGCGAGACCCTAGATATATACAATCGG
TTTAAAACTAAAACCGAGCGCATTATCGACCTTGGTGGTAACTATGTTACTCACGCCAAACAAGGTCGTTCTA
ATGTGCATTCCTGCTGTCCCATCCTTGACGTTAGGGACGGTGCTAGGCATACCGATCGATATATATCTTTAGCT
GCCTCTGTTGAAAATCGTCACAGAGAGTTACCGGTTGATTTCTGTTGCCATAAGTTCGAGGATTGTGGGGTC
AAGGCACCTTTTGCCATGGCTGTCCACTCCATCAGTGATATTCCTATCACTACGGTTGCTACACACTGCGTTAG
GCGCGGTGTGCGTAAGTTGATAGCTTCCGTCATGATGGACCCACTTATGATGCTTTATGACAAAGGTCACATA
CCCTTACTCAATGTTGATTGGGAAAAGGAAGACGTTGAATCGGGGAAAACCCTGATTCATTTCCATTTTGTT
GACGCACCCGGTTTAAGCTAYTCACACGATTTTAATACACTGTCCCAATACATGGTCACGAACCAAGTTATTG
TTAATAATTCCTACTCTTTTAGAGTAGAGAGGACAGCCTGTTTATCAGGTGTGTATATCGTGGAAATGACCCTG
TCTATGACAGATGGTCATTCTTTAGCTTATCTGAAGCCCATGCGTGATGTGTCATGCGCATGGTTGTCAAGTTT
GAGGAAGAAAGTTTTCGTCAAATTAGCCGTCCCTGTCAGTGCCGAGTGGTATGCTGAACARTTCGAGGTGC
GGTATGCGCTGATGGACGAATCCTTAGTTCGCTATGTTTCCGAAGCTGCCTTTCGGCAATTTTCGAAGACGA
AAGACCCTGAAACACTGGTTCAGTACATAGCAACTATGTTATCTTCCTCTTCAAATCATGTGGTCATTAACGGA
ATAACAATGCGAAGTGGTAGCCCAATCAAATTCGATGAATATGTTCCACTAGCTGTTACTTTTTACGTTATGGC
CGCTTGGCGTTACAAAATGATTGCCCCCGGTATCGATGCTGTGAAAACAAGAACCGAGAAGAACGTTGATG
CTCTTGATGAGAGGGGTCTTGTCAAGGAAGACTTTAATATCGTCAACGAATTTCTTGAGGAAGCTGGGTTAG
TTGGACCCAATTTGCCCCACCTCACGGATGTCATAAAAAATGCTGGACTCCGTTGTTTTGGGAAGAGGACCA
TCGAGAAAACCAGGGATGATGTTTCTTTAGTGAAACCGAGAAACTTGTTGCGTGAAGTGATCTACACGGTC
CGGAGTATCTTCGGGCTTACGATTTTGGACTCAGACTACAATTTGGTATCCGGTATACCTTCTCATATGAAGGC
AACTCATGTGTGGTCTGTATTCGTCGGAAATCTTGCATTCCCTGCATGCCTTAACGTCAATGAATGTGTTAATG
AGTTACTCGTTAATCACATGGAAATGGTAGAAGAGGCGAAGCAGGAAGAAACACGTCAACAAGCTTTTAAA
GATGCCAGAGATCGTGCTCTGATGACGATAGCGAAAGCTATCGAGAAGGATCAGACTGTGAAGGATGGTCT
GTTACCGATTCTTGATTTGTGTAAGATCAAAGAAGAATTGATGACGGCCTCGAATTCCTTGAGTTTAACTCCA
GAGGCCATTGAGAACACAGATTCAAGATTGATGAAAGCGTCCGGAAGCGACGTCAATCCTTATGCCGATTC
CATAAAGGAGGCCATACATTATTTTAATGATGTAGAAGTGGCCAACACAAGGAATCTTCGTAGTTTGGGAAC
TTATCTTGGATGGTCGATACCCAAGAATAAGCAAACATACGATGCCCTAAGGGGTAGGAATGAATCTGTCAG
GGTGTACGTACCTTATGAAAATAAGTGGTACCCTTCTGCACCCACCAGTCATTATGAAAGGGCTATGACTGTT
GATGGGTATGTTTCGCTTCAATGGAATTCTGAAGCGATTACCGACAAATGTAGACATGATTTGTCTAAATACC
ATGTCTTGGTTGTCGACGATTCATGCATTTTCTGTTCTGGCCAGAGAATGATCCCAGCTTTAGAAGCCGCTTT
AAAACTGGTCCCAACCTTTAAGATCACAATTGTTGATGGTGTCGCGGGTTGTGGTAAGACTACACATCTGAA
GAAGATAGCACGCATTGATGCAAGCGCTGCAGGTAGTCCTGATCTGGTGTTGACAAGTAATCGCAGTTCATC
AGATGAGCTTAAGGAAGTTATAGATTGTCCTGATGTGATGAAATACCGCATTAGAACGGTTGATAGCTACTTA
ATGCTCAAATCCTGGTTTTCAGCGGAGCGACYATTGTTTGATGAATGCTTCTTGACGCATGCGGGCTGCGTGT
ATGCAGCAGCCACTTTGGCTCAGGTGAAAGAAGTAATTGCTTTTGGTGACACGGAG------------
----------------------TTCGCATGGAACATCATAAGGTTGTTGGAAAAATTGACGTCCAAA
CAACGACCTATAGGTGTCCAAGAGATGCCACAGCTTGTTTAAAGAAATTTTTCTATAAAAACAAAACTGTGA
AGTCAGCGAGTGTCGTGGAACGCTCGCTTGAATTATGCCCAATTCAAAGTGTGATTCAAATTCAACCGGAGC
GTGATGTTTTGTACATGACACACACGCGAGCAGATAAGGAGACCCTGATGAGAATACCCGGGATGCCGAAA
GATAGAATTAAAACCACTCATGAAGCCCAAGGTGAAACCTGGGATCATGTGGTGATGTTTAGACTTTCGAAG

ACCACTAATCTGCTCCATTCGGGAAAAGGACCTGAATTAGGTCCATGTCACAATTTGGTTGCCATATCTAGAC
ATCGGAAATCATTTAGGTATTTTACTGTTTCACCTCATGATAACGATGATCAGATAGTGAAGTGTATCAGTTAC
GCCAGGTCCTTAAGTTCAGGGGACTTGGATGGGGTCCGTGTTTTAAACTGATGCAAGTTTTAGTGTACATCC
TCTATTGAGGTGCCGCAGGTTTCCTGCGATGATTTATATATATATATATATATTGCCAGTGGTTAGGCGGGTGTG
ATGCTTCTCCAACACACTTCCACTGATGC

>TSV RNA2 (A6-5 isolate)
AATCAGAACCTCCTGAAATATGGATTCCGTTTTAAAGAACCTCATTGTGTATCACTTGAGCAGACGAATTGAC
ATTGGCATGTCATTTGGTATTGAGCCGGCTGATTATGTTGATTGGGTTAAACTTTTCCTTATGAAGTTTATAATA
GAACATACAGCCAGGTTTGCCGACTTTGCCACCATTCATATGACAATGTTAATGGTGTTAGGCACAGAAGAT
GATGAATATGTGGAAAAGGACACTCCATTTGTGGAGATTGACCCCTTTTACCTTCCATATGATGATCTTGATGT
GGATTACACTTCATTACATGTGTTTGGTGATGAAAACCAATCCTGTTCCGACCGAGATGAGTTGAGTGACTTC
GTGTCGAACATCTCACACGTCCCCTAAGGTGTTTCTTGGGGTAGTGAATCAGATACCTCTTTCGTAGAGCATC
TTGAAGAAATTCAAGAGATACCTACGAAAGTCGTTTTTACTGATGAACCGGTTGAAGAAATTCCGTTTGACG
ATGATGGTAAAGTCGTTGACGATGTTTGGGTTGATGCCGAGCCTTCTAAAGCTCCGGAAATTTCCTGTGATG
CCGACATTCGCACTTGTGGTTTCGTTTCGTATGCCTCTGGAAGTTCCAAGGTGTCGAAATGGACACCGAAGG
TGAGTCAGGTTAAACCCGACCCTTCTGTAATCCAAGATGCAGTCGATGAGATTTTCCCCCACCATCATTCTGT
TGATGATAGATTCTTCCAAGAATGGGTTGAAACCCATGATATAGATCTGGAAGTCACGAGTTGTGATTTGGAC
TTGTCCACGTTTAATGACTGGACAAAAGGGATTGACACTCGATTGGTACCGAACTTGAATGTTGGGGGGTT
GTCCCACAGGGTCCCAACTCAGCGAGAAGCTTTGTTGGCTATAAAGAAGAGGAACATGAATGTTCCTGAAC
TTCAAAGCAGTTTTGACCACGATGATGTTTTAAACCGATGTGTTAATAGATTCATTACACATGTCGTTGATAAG
ACTCGTTTGTCAAAACTGAACCCGATATCTGGTGAGGAATTACATTACTTCAACCAGTATTTGGAAAATAAGA
ATCCGCCCTTAAGTGAATACAAGGGTCCGGTGCCATTGGTGGCTTTAGATAAGTATATGCACATGATAAAGAC
CACCTTGAAGCCAGTGGAAGAGGATAACCTCCACATAGAACGGCCAATTCCTGCTACAATCACTTATCATAA
GAAGGGTGTTGTTATGATGACGTCCCCTTATTTTTTGTGTGCAATGGTGAGGTTACTCTATGTGTTGAAATCA
AAATTTGTTGTTCCAACTGGAAAGTACCAC-------------------------------
----------AGGAATTCAAGGAAATCGATTTCTCAAAATTCGACAAATCTCAAGGTCGATTACATCACG
ATGTGCAGTTTAGACTGTTCCTAACTCTTGGTGTACCAGAGCATTTTGTGACCACATGGTTCAACTCCCATGA
AAGAAGTCACATTAGGGATCGTGATTGTGGAATCGGATTTTCCGTTGATTATCAACGAAGARCTGGTGATGC
ATGCACTTACTTAGGAAACACTCTTGTTACATTGAGTGTCCTTAGTTATGTGTATGATTTGTCCAATCCGAACAT
ATTGTTCGTTGCCGCAAGTGGCGATGACAGTCTTATTGGATCTAGAGAACCTTTACCGCGTGAAAAGGAAGA
TTTGTGCGTGTCCCTTTTCAATTTTGAAACGAAGTTTCCTCATAACCAACCATTCATTTGTTCAAAATTTTTGT
TGGTTGTTGAGTGCGATGATGGTTCGGAAGAAGTTTTGGCAGTTCCCAATCCTCTAAAGCTTCTCCAAAAA-
CTTGGACCTAAAAATCTTCAAGTCACAGTCTTAGATGATTATTACCAAAGTCTCTGTGATATATTGTGGGTTTT
TAATGATGCCGACATATGTCGAAGAACCGCTGAGTTGGCGGAATACAGACGTTTCAAAGGAACCAAGAAAT
GTCTGTTTCTGGAGTCCGCTCTGTTAAGCTTACCTAGCTTAGTGGCGAACAGGATGAAATTCATTCGTAGAAC
TGTCAATTTAGAAAGTTCTAAAGTTTGTATTCGTAACGATGTTTATTCCGATCTTGTTCCTCACTTTGACTCTCG
TGTCAGCAGATGTGATGACTCCAAAGGAGTTCGAATCTCAACCTCCGATGACGGAACATCATCCGAGTGTGC
CTCCGYTGGGTTCGGTAAAGCCGAATATCGCGGAGAGGCCCGGGGTAGAATTAAACCCCGGAGAGACCATA
AACGTGAAGGCGGAACAGTCCAATACAATCAATCCAGTGGAGTTGAAACTGGAAGAGCGAAGTCCTCCCG
GAAGGGTAGGATCAAATTGCATTGATTGTGCTATTTCCAACTTGCCGGAGGCTATGTTCTCCGTCAAGGTTCC
GAAGTTGAATATCAACTTCGAAGTGTCCGATTTTCCGTCCTCAAGGTTAATATTTGCCTCGTTAGCCCAAAGA
GTGAAGTCTATTCCCTTTATTGAATCTCTGAGTTTTCCCAGTGATATTCAAAGGATGCAACTTCGCGCATTGG
GTGACGTAGAAGTCCTCATCGCAATTCCAAAATATGGTTGGAAACAGATTTTGAAATTGTCTGATGTGGTTTC

CGGGTTTGATATCCCGAAGATCCCATCCATAGCTCCCAAGGTGGAGTCATGTGTTGGTGATTGTCTAAGTTCT
TGACTTAGATATTCCTCTTTTAAAGAGATGCCGCTTTATGCGATGGTGTCAGTGGATGGGCGAAACATGCGCT
TCTCTATGTTTCCCACT

>TSV RNA3 (A6-5 isolate)
AGCGRAAATACCACTTGCAATTTGATTCCGAATCGGACGATTTCCGACTTTGAATTCCTACAAGTCGAGACCA
TTTAGGTCGATCAAATTGCTCGTCATTAGAATACGACTGATTTCACAGAGGATCTTCTGAATACAGTTATCCCG
TTGACTCAGTTGCTGTTCGGGCTGTGAAATTCAGGACCCGCCACTAAAAAGGAAGATGGCGTTAGTACCAA
CGATGAAAGCTTTAACATTCTCTGCAGAAGATGAGATTTCCCTCGAGAAGGCTATAACCGAAGCACTTTCTG
GTTCCGTTGAGATTAACATGGGTCTACGACGTTGCGCAGCTTTCCCTGCTGTTAACACAGGTGCTTTCCTGTG
TGAGTTGACAACCAAAGAGACGAAATCCTTTATTGGTAAATTTTCTGATAAAGTTCGAGGACGTGTCTTTGT
AGATCACGCGGTTATTCATTTGATGTATATACCCGTGATATTGAACACCACACATGCCATCGCAGAGCTAAAAT
TGAAAAACTTAGCTACCGGTGATGAACTTTATGGTGGTACGAAAGTCAACTTGAGCAAGGCCTTCATTTTAA
CGATGACTTGGCCTCGCTCATTATTTGCTGAAGCAGTTCATTCCCACAGAGGATTATACCTGGGGGGAACTG
TTTCCTGCGCTTCCTCAGTGCCTGCAAACGCCAAAATTGGGATGTGGTACCCCATGTGGTCGGAAAAGGTTT
CGAATAAACAACTGTATCAAAATACAGTTAATATTCATAATACCGAAGCACTTGAGACGTTTACGCGGACGAT
GATCAGCAGTGACAGGGAAATGAGATCATTATTGAGAAGTCGTGCCTCAATTGATATTGCTGCGAAGACGTT
TGAGAAACCCGTGATATGCTCGGAAC———————————————————————————————————
————————————————————————————CCGATAAGGACGATGACGCAGGAACGTCAATTTTA
GGACCTAAAATGGTCCCAATTGAACAAGTACCGTCGGTTAAAGTTCCATCGTCGGAAGCAGGTAGGAACCT
GCTTTCAGCCTGATTGTTGGGTTGTGTAAGACATGGGGGGCTTTGAGTAAAGGGGCTAGTCCYCCAGTGTG
AGACGAGTATTAAGTGGATGAATTCTAGAAATAGATAAGTCGCTTCTCGGACTTACCTGGGATGTATGAATAC
TTTGATCCAAAGTCCAGACCATCCATCCAACGCCATGTCTTCTCGTACTAACAACCGCTCAAACAACAGCAGA
TGCCCAACTTGTGTTGACGAGTTGGAAGCTTTGGCGAGGAATTGTCCCGCCCATATTCCCGCGAACACTGTG
TCGCGACGTCAGCGGCGTAATGCCGCTAGGGCTGCTGCGTATCGAAACGCGAACGCCCGTCTACCAGTACC
GATTCCAGTGGTGCCGGTTTCCCGCCCTCAAGCGAAGGCGTCGTT-GAGGTTGCCCAATAATCAAGTTTGGG
TAACTCGCAAAGCGAGTGAATGGTCTGCAAAGACCGTTGATACCAACGATGCTATCCCCTTCAAAACCATAG
TCGAGGGGATTCCCGAAATCAATGATGAGACGAAGTTTTTCCGTCTCTTAATTGGTTTTGTCGCCGTCTCTGA
TGGGACGTTTGGGATGGTTGATGGAGTAACAGGCGATGTTATTCCTGACCCACCGGTCGTTGGACGGTTGG
GTTTCAAGAAGAATACCTACCGCAGCCGAGATTTTGATCTCGGTGGTAAACTTCTCAACCAACTAGACGACA
GAGCTGTCGTCTGGTGCCTCGACGAAAGACGTCGAGATGCCAAGAGGGTTCAGCTGGCAGGTTATTGGATT
GCCATTTCCAAACCAGCTCCCTTGATGCCACCAGAAGATTTTCTGGTGAATCAAGATTGACTAGATGGTCACC
TCGGTAGGACCGAGTTGCCGCACATTAGAAGAAATTCCTGTGTGCGATGTGGGTTTACACCACCACGTATTG
TAAATCAATACGTTTGGTCGTGTAACATGTGTTCCTATGAACAACATGCATAGCTGCCGCGCATCCGGGCGTG
AGTCTATGATCCATGCAAGCATTCCGAATCGGACGATTTCCATGGGAAACSATGAGACCTTTAAGGTCGATGC
TTGCGTTTGGTACCAGTAGTATATAAATACTACTGATGCCTCCTTTTAGGAG
Note: "—" represents gap.
