# Supplementary material for: Molecular Characterization of an Isolate of Tobacco Streak Virus Naturally Infecting Areca catechu L. in China—A First Case in the Family Arecaceae
Source: Plants (Basel). 2026 Jun 16;15(12):1864. doi: 10.3390/plants15121864 (PMC13306292; doi:10.3390/plants15121864)
Supplement: Supplementary file 1 [file plants-15-01864-s001.zip › Table S1 List of tobacco streak viruses and strawberry necrotic shock virus information from different plant host sources.pdf]

Table S1 List of tobacco streak viruses and strawberry necrotic shock virus information from different plant host sources.

Isolate	GenBank	Segment	Country	Host	Chinese name
A6-5	PZ244272	RNA1	China: Sanya	*Areca catechu* L: areca-nut	槟榔
	PZ244273	RNA2			
	PZ244274	RNA3			
DSMZ PV-0612	ON924217.1	RNA1	Germany	*Rudbeckia*: coneflowers	金光菊
	ON924218.1	RNA2			
	ON924219.1	RNA3			
dp	KR017708.1	RNA1	USA	*Dahlia pinnata*: garden dahlia	大丽花
	KR017709.1	RNA2			
	KR017710.1	RNA3			
TSV-KN	PX393120.1	RNA1	India: Karimnagar, Telangana	*Helianthus annuus*: Sunflower	向日葵
	PX393121.1	RNA2			
	PX393122.1	RNA3			
PHAP15	KY176874.1	RNA1	India	*Parthenium hysterophorus:* Parthenium weed	银胶菊
	KY176875.1	RNA2			
	KY176876.1	RNA3			
Nikita	PV637734.1	RNA1	Russia	*Ficus carica*: Common fig	无花果
	PV637735.1	RNA2			
	PV637736.1	RNA3			
CUKA13	KY176871.1	RNA1	India: Karnataka, Tumkur	*Cucumis sativus*: Cucumber	黄瓜
	KY176872.1	RNA2			
	KY176873.1	RNA3			
SFAP17	KY176868.1	RNA1	India: Andhra Pradesh, Kurnool	*Helianthus annuus*: Sunflower	向日葵
	KY176869.1	RNA2			
	KY176870.1	RNA3			
FL13-07	KM504246.1	RNA1	USA	*Cucurbita pepo* L.: Zucchini	西葫芦
	KM504247.1	RNA2			
	KM504248.1	RNA3			
okra	FJ561302.1	RNA1	India: Tamilnadu	*Abelmoschus esculentus*: okra	秋葵
	FJ561303.1	RNA2			
	FJ561304.1	RNA3			
pumpkin	FJ561299.1	RNA1	India: Bangalore	*Cucurbita pepo*: pumpkin	南瓜
	FJ561300.1	RNA2			
	FJ561301.1	RNA3			
TSV-BAN	PX393125.1	RNA1	India: Bangalore, Karnataka	*Helianthus annuus*: Sunflower	向日葵
	PX393124.1	RNA2			
	PX393123.1	RNA3			
TSV-CBE	PX393119.1	RNA1	India: Coimbatore, Tamil Nadu	*Helianthus annuus*: Sunflower	向日葵
	PX393118.1	RNA2			
	PX354930.1	RNA3			

Henry	JX073656.1	RNA1	USA	*Nicotiana tabacum*: tobacco	烟草
	JX073657.1	RNA2			
	JX073658.1	RNA3			
Illinois	FJ403375.1	RNA1	USA	*Glycine max*: Soybean	大豆
	FJ403376.1	RNA2			
	FJ403377.1	RNA3			
OH-2011	KT445967.1	RNA1	USA	*Glycine max*: Soybean	大豆
	KT445968.1	RNA2			
	KT445969.1	RNA3			
TSV-NDY	PX120834.1	RNA1	India: Nandhiyal, Andhra Pradesh	*Helianthus annuus*: Sunflower	向日葵
	PX120833.1	RNA2			
	PX120832.1	RNA3			
TSV-CHK	PV784943.1	RNA1	India: Karnataka, Chikkabalapura	*Tagetes*: Marigold	万寿菊
	PV784944.1	RNA2			
	PV784945.1	RNA3			
TSV-PxBG2	PV525001.1	RNA1	Russia	*Phlox paniculata*: Garden Phlox	天蓝绣球
	PV525002.1	RNA2			
	PV525003.1	RNA3			
TSV-Px31	PV524998.1	RNA1	Russia	*Phlox paniculata*: Garden Phlox	天蓝绣球
	PV524999.1	RNA2			
	PV525000.1	RNA3			
TSV-Px15	PV524995.1	RNA1	Russia	*Phlox paniculata*: Garden Phlox	天蓝绣球
	PV524996.1	RNA2			
	PV524997.1	RNA3			
Uga-Westn	OM802497.1	RNA1	Uganda	*Cucurbita pepo*: pumpkin	南瓜
	OM802498.1	RNA2			
	OM802499.1	RNA3			
IN:Kar:Cucumber:21	OQ514052.1	RNA1	India	*Cucumis sativus*: Cucumber	黄瓜
	OQ514053.1	RNA2			
	OQ514054.1	RNA3			
DSMZ PV-0738	PP503015.1	RNA1	USA: Wisconsin	*Trifolium repens*: white clover	白车轴草
	PP503016.1	RNA2			
	PP503017.1	RNA3			
BR	MT360267.1	RNA1	Brazil	*Glycine max*: Soybean	大豆
	MT360268.1	RNA2			
	MT360269.1	RNA3			
IA-3-2017	MT669383.1	RNA1	USA	*Glycine max*: Soybean	大豆
	MT669384.1	RNA2			
	MT669385.1	RNA3			
MD-2016	MT669377.1	RNA1	USA	*Glycine max*: Soybean	大豆
	MT669378.1	RNA2			
	MT669379.1	RNA3			
2334	JX463337.1	RNA1	Australia	*Verbesina*	金冠菊

	JX463338.1	RNA2		*encelioides*: golden crownbeard	
	JX463339.1	RNA3			
1973	JX463334.1	RNA1	Australia	*Helianthus annuus* cv. Sunbird 7: Sunflower	向日葵
	JX463335.1	RNA2			
	JX463336.1	RNA3			
TSV CNB	OR183775.1	RNA1	China	*Echinacea purpurea*: purple coneflower	松果菊
	OR183776.1	RNA2			
	OR183777.1	RNA3			
TSV-HYD	PX120817.1	RNA1	India: Hyderabad, Telangana	*Helianthus annuus*: Sunflower	向日葵
	PX120818.1	RNA2			
	PX120819.1	RNA3			
cladodes	PX788478.1	RNA1	India	*Selenicereus undatus*: dragon fruit	火龙果
	PX788480.1	RNA2			
	PX788479.1	RNA3			
Strawberry necrotic shock virus (SNSV)	NC_008708.2	RNA1	USA: Maryland	*Fragaria* spp.: strawberry	草莓
	NC_008707.1	RNA2			
	NC_008706.1	RNA3			
